# Supplementary material for: Comparison of kidney allograft survival in the Eurotransplant senior program after changing the allocation criteria in 2010—A single center experience
Source: PLoS One. 2020 Jul 23;15(7):e0235680. doi: 10.1371/journal.pone.0235680 (PMC7377418; doi:10.1371/journal.pone.0235680)
Supplement: Attachment — Submitted filename: Response To Reviewers_2.docx [file pone.0235680.s002.docx]

|  |  |
| --- | --- |
| Prof. Dr. med. Stefan Reuter **∙** Universitätsklinikum Münster **∙** Medizinische Klinik **∙** Albert-Schweitzer-Campus 1, Gebäude A1 **∙** 48149 Münster  **Dr. Gołębiewska, Academic Editor PLOS ONE** | **Medizinische Klinik D, Allgemeine Innere Medizin und Notaufnahme sowie Nieren- und Hochdruckkrankheiten und Rheumatologie**  Direktor  Univ. Prof. Dr. med. Hermann-Joseph Pavenstädt  **Prof. Dr. med. Stefan Reuter**  Albert-Schweitzer-Campus 1, Gebäude A1  Anfahrtsadresse: Waldeyerstraße 1  48149 Münster  T +49 251 83-50607  F +49 251 83-45813  Stefan.Reuter@ukmuenster.de  www.klinikum.uni-muenster.de |

Münster, 10.06.2020

Comparison of Kidney Allograft Survival in the Eurotransplant Senior Program after Changing the Allocation Criteria in 2010 – A Single Center Experience” (**PONE-D-20-01629R1**)

Dear Dr. Gołębiewska,

Comparison of Kidney Allograft Survival in the Eurotransplant Senior Program after Changing the Allocation Criteria in 2010 – A Single Center Experience

PONE-D-20-01629R1

Dear Dr. Gołębiewska,

Thank you for considering our revised manuscript entitled: “Comparison of Kidney Allograft Survival in the Eurotransplant Senior Program after Changing the Allocation Criteria in 2010 – A Single Center Experience” (**PONE-D-20-01629R1**) for re-submission at *PLOS ONE*.

Firstly, we would like to thank you, and the reviewers for reading through our manuscript and for the excellent and helpful remarks. We appreciate the editor’s and reviewers’ comments and welcome the opportunity to address these queries and concerns in our revised manuscript. We have addressed all the editor’s and reviewers’ comments in our responses (as per below).

We now re-submit to you a revised version of our study, as well as this cover letter, which answers the reviewers’ questions and criticisms in a point-by-point discussion. The changes made in the revised manuscript are highlighted in yellow for clarity.

We hope that the revised manuscript, together with this point-by-point discussion will now answer all questions and make this revised manuscript suitable for publication in *PLOS ONE*.

Yours Sincerely,

Stefan Reuter

Reviewer #1: I have reviewed the revised version of the manuscript by Mehdorn et al and believe that the authors have improved the manuscript significantly. They have addressed all my remarks, although I still have some minor suggestions for further improvement of the manuscript.

1) Statistics (line 139-141). The authors have tried to describe how their multivariable models were built: “For multivariable model building, variables with a p-value of the likelihood ratio test > 0.1 were excluded and only variables with a p-value less than 0.05 were further analyzed by multivariable regression.” I´m not sure that I understand what the authors mean. What about variables with a p-value between 0.05 and 0.1? If they were included in the multivariable models (as I believe they should), it should have been described as: “For multivariable model building, variables with a p-value of the likelihood ratio test > 0.1 were excluded and only variables with a p-value less than 0.1 were further analyzed by multivariable regression.” Please revise.

We thank the reviewer for this helpful comment and agree that our formulation was difficult to understand. We have modified the paragraph accordingly as the reviewer was fully correct.

**Old:**

For multivariable model building, variables with a p-value of the likelihood ratio test > 0.1 were excluded and only variables with a p-value less than 0.05 were further analyzed by multivariable regression.

**New:**

For multivariable model building, variables with a p-value of the likelihood ratio test > 0.1 were excluded and only variables with a p-value less than 0.1 were further analyzed by multivariable regression.

2) End points (line 120-121). The limitations of the eGFR should not be mentioned in the methods section but rather in the discussion (where it is actually discussed – line 284-287). This sentence should consequently be removed from the endpoint paragraph of the method section

We thank the reviewer for this valuable comment and have removed the sentence from the methods sections, but emphasized this point in the limitations section.

**Old:**

The primary endpoint was graft function using serum creatinine and estimated glomerular filtration rate (eGFR) calculated according Modification of Diet in Renal Disease Study Group (MDRD) as surrogates [1]. Graft function was chosen as primary endpoint due to the small number of patients included. However, we are aware of the limitations of correlating graft function according to MDRD, as creatinine levels depend on dietary intake and muscle mass, among other factors.

**New:**

The primary endpoint was graft function using serum creatinine and estimated glomerular filtration rate (eGFR) calculated according Modification of Diet in Renal Disease Study Group (MDRD) as surrogates [1]. Graft function was chosen as primary endpoint due to the small number of patients included.

3) Comorbidities. I accept the authors clarification of why they chose the specific comorbidities for their analyses. However, even if these comorbidities are the most common comorbidities in CKD and development of renal disease. However, they were included in multivariable models as explanatory variable for patient- and graft survival which may be associated with other variables. I think the fact that there are several unknown confounders, as for example other comorbidities, should be mentioned in the limitation section of the discussion.

We thank the reviewer for bringing up this point and fully agree with the point made. We apologize for not having mentioned this before.

**Old:**

Unfortunately, we have not found a better way to compare both cohorts.

**New:**

Unfortunately, we have not found a better way to compare both cohorts. We have included the most common comorbidities in CKD-patients in order to analyze their influence on graft outcome. However, other comorbidities and other unknown confounders not included in our analysis may also influence outcome after RRT and RTx.

4) Table 5,6 and 7. I would still prefer to have the p-values of all variables included in the multivariable model, no matter if they are significant or not.

We acknowledge that the reviewer is correct and that the missing data should be included in the final manuskript. We have modified the tables accordingly.

**Old:**

Table 5

| **Cox regression model for 5-year graft survival** | | | | |
| --- | --- | --- | --- | --- |
| **Parameters** | **Univariate** | | **Multivariable** | |
|  | **HR (95% CI)** | **p-value** | **HR (95% CI)** | **p-value** |
| **HC vs nESPC** | 2.094 (0.831 – 5.278) | 0.117 |  | |
| **Recipient age (years)** | 0.953 (0.866 – 1.050) | 0.331 |  | |
| **Recipient sex (male vs female)** | 1.229 (0.702 – 2.150) | 0.471 |  | |
| **Recipient BMI (>25 kg/m^2^)** | 2.304 (1.071 – 4.958) | **0.033** | 0.228 (0.036 – 0.288) | **0.012** |
| **Time of RRT (months)** | 1.012 (0.991 – 1.034) | 0.274 |  | |
| **Arterial hypertension recipient** | 0.759 (0.368 – 1.564) | 0.455 |  | |
| **Diabetes recipient** | 1.722 (0.846 – 3.504) | 0.134 |  | |
| **Hyperlipidemia recipient** | 0.877 (0.423 – 1.820) | 0.725 |  | |
| **Arteriosclerosis recipient** | 1.972 (1.059 – 3.701) | **0.035** |  | |
| **Cold ischemia time (hours)** | 0.930 (0.860 – 1.005) | 0.066 |  | |
| **Sum of HLA mismatches** | 0.738 (0.562 – 0.970) | **0.029** |  | |
| **Operation time (min)** | 1.000 (1.000 – 1.000) | **0.018** |  | |
| **Warm ischemia time (minutes)** | 0.985 (0.961 – 1.010) | 0.235 |  | |
| **Intraoperative complications (yes vs no)** | 1.615 (0.638 – 4.091) | 0.312 |  | |
| **Postoperative complications (yes vs no)** | 1.357 (0.764 – 2.411) | 0.297 |  | |
| **Length of hospital stay (d)** | 0.991 (0.974 – 1.009) | 0.320 |  | |
|  |  |  |  | |
| **Donor age (years)** | 1.011 (0.955 – 1.071) | 0.699 |  | |
| **Donor gender (male vs female)** | 1.402 (0.743 – 2.645) | 0.297 |  | |
| **Donor BMI (kg/m^2^)** | 1.046 (0.966 – 1.134) | 0.270 |  | |
| **Donor creatinine pre-procurement (mg/dl)** | 0.894 (0.442 – 1.809) | 0.756 |  | |
| **Arteriell Hypertension Donor** | 1.314 (0.671 – 2.576) | 0.426 |  | |
| **Diabetes Donor** | 1.168 (0.528 – 2.582) | 0.701 |  | |
| **Arteriosclerosis Donor** | 0.748 (0.385 – 1.453) | 0.391 |  | |
| **Hyperlipidemia Donor** | 0.614 (0.211 – 1.788) | 0.371 |  | |
| **Length of Donor on Intensive Care Unit (d)** | 1.090 (1.023 – 1.161) | **0.007** |  | |
|  |  |  |  | |
| **PF (yes vs no)** | 0.075 (0.018 – 0.308) | **< 0.001** |  | |
| **Biopsy-proven Rejection (yes vs no)** | 0.826 (0.512 – 1.335) | 0.435 |  | |
| **Intraoperative comlications (yes vs no)** | 1.235 (0.662 – 2.304) | 0.507 |  | |
| **Surgical complications (yes vs no)** | 0.844 (0.569 – 1.252) | 0.401 |  | |
| **Non-surgical complications (yes vs no)** | 0.716 (0.486 – 1.057) | 0.093 |  | |

**Table 5. Cox regression model for 5-year graft survival**

HR = hazard ratios, CI = 95% confidence interval. HC = historic cohort, ESP = European Senior Program, BMI = body mass index, HLA = human leukocyte antigen, PF = primary function

**New:**

Table 5

| **Cox regression model for 5-year graft survival** | | | | |
| --- | --- | --- | --- | --- |
| **Parameters** | **Univariate** | | **Multivariable** | |
|  | **HR**  **(95% CI)** | **p-value** | **HR**  **(95% CI)** | **p-value** |
| **HC vs nESPC** | 2.094  (0.831 – 5.278) | 0.117 |  | |
| **Recipient age**  **(years)** | 0.953  (0.866 – 1.050) | 0.331 |  | |
| **Recipient sex**  **(male vs female)** | 1.229  (0.702 – 2.150) | 0.471 |  | |
| **Recipient BMI**  **(>25 kg/m^2^)** | 2.304  (1.071 – 4.958) | **0.033** | 1.813  (1.175 – 2.799) | **0.012** |
| **Time of RRT**  **(months)** | 1.012  (0.991 – 1.034) | 0.274 |  | |
| **Arterial hypertension recipient** | 0.759  (0.368 – 1.564) | 0.455 |  | |
| **Diabetes recipient** | 1.722  (0.846 – 3.504) | 0.134 |  | |
| **Hyperlipidemia recipient** | 0.877  (0.423 – 1.820) | 0.725 |  | |
| **Arteriosclerosis recipient** | 1.972  (1.059 – 3.701) | **0.035** | 1.371  (0.886 – 2.121) | 0.929 |
| **Cold ischemia time**  **(hours)** | 0.930  (0.860 – 1.005) | 0.066 |  | |
| **Sum of HLA mismatches** | 0.738  (0.562 – 0.970) | **0.029** | 0.968  (0.811 – 1.155) | 0.449 |
| **Operation time**  **(min)** | 1.000  (1.000 – 1.000) | **0.018** | 1.000  (1.000 – 1.000) | 0.455 |
| **Warm ischemia time**  **(min)** | 0.985  (0.961 – 1.010) | 0.235 |  | |
| **Intraoperative complications (yes vs no)** | 1.615  (0.638 – 4.091) | 0.312 |  | |
| **Postoperative complications (yes vs no)** | 1.357  (0.764 – 2.411) | 0.297 |  | |
| **Length of hospital stay**  **(d)** | 0.991  (0.974 – 1.009) | 0.320 |  | |
|  |  |  |  | |
| **Donor age**  **(years)** | 1.011  (0.955 – 1.071) | 0.699 |  | |
| **Donor gender**  **(male vs female)** | 1.402  (0.743 – 2.645) | 0.297 |  | |
| **Donor BMI**  **(kg/m^2^)** | 1.046  (0.966 – 1.134) | 0.270 |  | |
| **Donor creatinine pre-procurement (mg/dl)** | 0.894  (0.442 – 1.809) | 0.756 |  | |
| **Arteriell Hypertension Donor** | 1.314  (0.671 – 2.576) | 0.426 |  | |
| **Diabetes Donor** | 1.168  (0.528 – 2.582) | 0.701 |  | |
| **Arteriosclerosis Donor** | 0.748  (0.385 – 1.453) | 0.391 |  | |
| **Hyperlipidemia Donor** | 0.614  (0.211 – 1.788) | 0.371 |  | |
| **Length of Donor on Intensive Care Unit (d)** | 1.090  (1.023 – 1.161) | **0.007** | 1.046  (0.998 – 1.096) | 0.221 |
|  |  |  |  | |
| **PF**  **(yes vs no)** | 0.075  (0.018 – 0.308) | **< 0.001** | 1.400  (0.917 – 2.135) | 0.870 |
| **Biopsy-proven Rejection**  **(yes vs no)** | 0.826  (0.512 – 1.335) | 0.435 |  | |
| **Intraoperative complications**  **(yes vs no)** | 1.235  (0.662 – 2.304) | 0.507 |  | |
| **Surgical complications**  **(yes vs no)** | 0.844  (0.569 – 1.252) | 0.401 |  | |
| **Non-surgical complications**  **(yes vs no)** | 0.716  (0.486 – 1.057) | 0.093 |  | |

**Table 5. Cox regression model for 5-year graft survival**

HR = hazard ratios, CI = 95% confidence interval. HC = historic cohort, ESP = European Senior Program, BMI = body mass index, HLA = human leukocyte antigen, PF = primary function

**Old:**

Table 6

| **Cox regression model for 5-year death-censored graft survival** | | | | |
| --- | --- | --- | --- | --- |
| **Parameters** | **Univariate** | | **Multivariable** | |
|  | **HR (95% CI)** | **p-value** | **HR (95% CI)** | **p-value** |
| **HC vs nESPC** | 2.575 (1.016 – 6.527) | **0.046** |  | |
| **Recipient age (years)** | 0.937 (0.848 – 1.035) | 0.197 |  | |
| **Recipient sex (male vs female)** | 1.739 (0.980 – 3.085) | 0.058 |  | |
| **Recipient BMI (kg/m^2^)** | 2.199 (0.994 – 4.862) | 0.052 |  | |
| **Time on dialysis (months)** | 1.001 (0.989 – 1.034) | 0.342 |  | |
| **Arterial hypertension recipient** | 0.647 (0.315 – 1.330) | 0.236 |  | |
| **Diabetes recipient** | 2.300 (1.119 – 4.731) | **0.024** | 0.0642 (0.336 – 1.311) | **0.002** |
| **Hyperlipidemia recipient** | 0.790 (0.372 – 1.681) | 0.514 |  | |
| **Arteriosclerosis recipient** | 2.389 (1.246 – 4.580) | **0.009** |  | |
| **Cold ischemia time (hours)** | 0.947 (0.878 – 1.021) | 0.155 |  | |
| **Sum of HLA mismatches** | 0.744 (0.567 – 0.975) | **0.032** |  | |
| **Operation time (min)** | 1.000 (1.000 – 1.000) | 0.097 |  | |
| **Warm ischemia time (minutes)** | 0.982 (0.957 – 1.008) | 0.176 |  | |
| **Intraoperative complications (yes vs no)** | 1.943 (0.730 – 5.172) | 0.184 |  | |
| **Postoperative complications (yes vs no)** | 1.227 (0.698 – 2.159) | 0.478 |  | |
| **Length of hospital stay (days)** | 0.999 (0.980 – 1.018) | 0.918 |  | |
|  |  |  |  | |
| **Donor age (years)** | 1.011 (0.953 – 1.072) | 0.726 |  | |
| **Donor gender (male vs female)** | 1.202 (0.611 – 2.365) | 0.595 |  | |
| **Donor BMI (kg/m^2^)** | 1.050 (0.967 – 1.139) | 0.245 |  | |
| **Donor creatinine pre-procurement (mg/dl)** | 2.575 (1.016 – 6.527) | 0.372 |  | |
| **Arteriell Hypertension Donor** | 1.178 (0.599 – 2.317) | 0.643 |  | |
| **Diabetes Donor** | 0.882 (0.377 – 2.065) | 0.773 |  | |
| **Hyperlipidemia Donor** | 0.763 (0.260 – 2.233) | 0.621 |  | |
| **Arteriosclerosis Donor** | 0.666 (0.347 – 1.277) | 0.221 |  | |
| **Length of Stay on Intensive Care Unit (donor) (d)** | 1.062 (0.997 – 1.132) | 0.062 |  | |
|  |  |  |  | |
| **PF (yes vs no)** | 0.098 (0.022 – 0.437) | **0.002** |  | |
| **Biopsy-proven rejection (yes vs no)** | 0.695 (0.424 – 1.139) | 0.149 |  | |
| **Intraoperative comlications (yes vs no)** | 0.536 (0.240 – 1.195) | 0.127 |  | |
| **Surgical complications (yes vs no)** | 0.800 (0.436 – 1.469) | 0.472 |  | |
| **Non-surgical complications (yes vs no)** | 0.728 (0.406 – 1.304) | 0.286 |  | |

**Table 6. Cox regression model for 5-year death-censored graft survival**

HR = hazard ratios, CI = 95% confidence interval. HC = historic cohort, ESP = European Senior Program, BMI = body mass index, HLA = human leukocyte antigen, PF = primary function

**New:**

Table 6

| **Cox regression model for 5-year death-censored graft survival** | | | | |
| --- | --- | --- | --- | --- |
| **Parameters** | **Univariate** | | **Multivariable** | |
|  | **HR**  **(95% CI)** | **p-value** | **HR**  **(95% CI)** | **p-value** |
| **HC vs nESPC** | 2.575  (1.016 – 6.527) | **0.046** | 1.210  (0.822 – 1.780) | 0.276 |
| **Recipient age**  **(years)** | 0.937  (0.848 – 1.035) | 0.197 |  | |
| **Recipient sex**  **(male vs female)** | 1.739  (0.980 – 3.085) | 0.058 |  | |
| **Recipient BMI**  **(kg/m^2^)** | 2.199  (0.994 – 4.862) | 0.052 |  | |
| **Time on dialysis**  **(months)** | 1.001  (0.989 – 1.034) | 0.342 |  | |
| **Arterial hypertension recipient** | 0.647  (0.315 – 1.330) | 0.236 |  | |
| **Diabetes recipient** | 2.300  (1.119 – 4.731) | **0.024** | 1.514  (0.983 – 2.331) | 0.403 |
| **Hyperlipidemia recipient** | 0.790  (0.372 – 1.681) | 0.514 |  | |
| **Arteriosclerosis recipient** | 2.389  (1.246 – 4.580) | **0.009** | 1.318  (0.889 – 1.956) | 0.257 |
| **Cold ischemia time**  **(hours)** | 0.947  (0.878 – 1.021) | 0.155 |  | |
| **Sum of HLA mismatches** | 0.744  (0.567 – 0.975) | **0.032** | 0.925  (0.795 – 1.078) | 0.059 |
| **Operation time**  **(min)** | 1.000  (1.000 – 1.000) | 0.097 |  | |
| **Warm ischemia time**  **(min)** | 0.982  (0.957 – 1.008) | 0.176 |  | |
| **Intraoperative complications (yes vs no)** | 1.943  (0.730 – 5.172) | 0.184 |  | |
| **Postoperative complications (yes vs no)** | 1.227  (0.698 – 2.159) | 0.478 |  | |
| **Length of hospital stay**  **(days)** | 0.999  (0.980 – 1.018) | 0.918 |  | |
|  |  |  |  | |
| **Donor age**  **(years)** | 1.011  (0.953 – 1.072) | 0.726 |  | |
| **Donor gender**  **(male vs female)** | 1.202  (0.611 – 2.365) | 0.595 |  | |
| **Donor BMI**  **(kg/m^2^)** | 1.050  (0.967 – 1.139) | 0.245 |  | |
| **Donor creatinine pre-procurement (mg/dl)** | 2.575  (1.016 – 6.527) | 0.372 |  | |
| **Arteriell Hypertension Donor** | 1.178  (0.599 – 2.317) | 0.643 |  | |
| **Diabetes Donor** | 0.882  (0.377 – 2.065) | 0.773 |  | |
| **Hyperlipidemia Donor** | 0.763  (0.260 – 2.233) | 0.621 |  | |
| **Arteriosclerosis Donor** | 0.666  (0.347 – 1.277) | 0.221 |  | |
| **Length of Stay on Intensive Care Unit (donor) (d)** | 1.062  (0.997 – 1.132) | 0.062 |  | |
|  |  |  |  | |
| **PF**  **(yes vs no)** | 0.098  (0.022 – 0.437) | **0.002** | 1.596  (1.081 – 2.356) | 0.622 |
| **Biopsy-proven rejection**  **(yes vs no)** | 0.695  (0.424 – 1.139) | 0.149 |  | |
| **Intraoperative complications (yes vs no)** | 0.536  (0.240 – 1.195) | 0.127 |  | |
| **Surgical complications**  **(yes vs no)** | 0.800  (0.436 – 1.469) | 0.472 |  | |
| **Non-surgical complications**  **(yes vs no)** | 0.728  (0.406 – 1.304) | 0.286 |  | |

**Table 6. Cox regression model for 5-year death-censored graft survival**

HR = hazard ratios, CI = 95% confidence interval. HC = historic cohort, ESP = European Senior Program, BMI = body mass index, HLA = human leukocyte antigen, PF = primary function

**Old:**

Table 7

| **Cox regression model for 5-year patient survival** | | | | |
| --- | --- | --- | --- | --- |
| **Parameters** | **Univariate** | | **Multivariable** | |
|  | **HR (95% CI)** | **p-value** | **HR (95% CI)** | **p-value** |
| **HC vs nESPC** | 1.829 (0.734 – 4.557) | 0.195 |  | |
| **Recipient age (years)** | 0.955 (0.870 – 1.049) | 0.337 |  | |
| **Recipient sex (male vs female)** | 2.100 (1.144 – 3.855) | **0.017** |  | |
| **Recipient BMI (<25 kg/m^2^)** | 2.225 (0.984 – 5.032) | 0.055 |  | |
| **Time on dialysis (months)** | 1.012 (0.990 – 1.035) | 0.287 |  | |
| **Arterial hypertension recipient** | 0.617 (0.289 – 1.319) | 0.213 |  | |
| **Diabetes recipient** | 2.671 (1.311 – 5.442) | **0.007** |  | |
| **Hyperlipidemia recipient** | 0.989 (0.476 – 2.503) | 0.976 |  | |
| **Arteriosclerosis recipient** | 3.152 (1.620 – 6.132) | **0.001** |  | |
| **Cold ischemia time (hours)** | 0.911 (0.840 – 0.988) | **0.025** |  | |
| **Sum of HLA mismatches** | 0.830 (0.636 – 1.085) | 0.173 |  | |
| **Operation time (min)** | 1.000 (1.000 – 1.000) | **0.010** |  | |
| **Warm ischemia time (minutes)** | 0.987 (0.961 – 1.013) | 0.310 |  | |
| **Intraoperative complications (yes vs no)** | 2.212 (0.823 – 5.945) | 0.116 |  | |
| **Postoperative complications (yes vs no)** | 1.134 (0.652 – 1.972) | 0.656 |  | |
| **Length of hospital stay (days)** | 1.005 (0.986 – 1.025) | 0.606 |  | |
|  |  |  |  | |
| **Donor age (years)** | 0.976 (0.920 – 1.036) | 0.432 |  | |
| **Donor gender (male vs female)** | 1.113 (0.547 – 2.266) | 0.767 |  | |
| **Donor BMI (kg/m^2^)** | 0.988 (0.912 – 1.069) | 0.761 |  | |
| **Donor creatinine pre-procurement (mg/dl)** | 1.118 (0.533 – 2.345) | 0.769 |  | |
| **Arteriell Hypertension Donor** | 1.310 (0.689 – 2.489) | 0.410 |  | |
| **Diabetes Donor** | 1.404 (0.635 – 3.106) | 0.402 |  | |
| **Hyperlipidemia Donor** | 2.157 (0.795 – 5.851) | 0.131 |  | |
| **Arteriosclerosis Donor** | 0.793 (0.405 – 1.553) | 0.499 |  | |
| **Length of Donor on Intensive Care Unit (d)** | 1.083 (1.017 – 1.153) | **0.013** |  | |
|  |  |  |  | |
| **PF (yes vs no)** | 0.272 (0.065– 1.143) | 0.076 |  | |
| **Biopsy-proven rejection (yes vs no)** | 0.707 (0.419 – 1.193) | 0.194 |  | |
| **Intraoperative complications (yes vs no)** | 0.572 (0.297 – 1.102) | 0.095 |  | |
| **Postoperative surgical complications (yes vs no)** | 0.954 (0.634 – 1.436) | 0.822 |  | |
| **Non-surgical complications (yes vs no)** | 0.510 (0.338 – 0.769) | **<0.001** | 0.198 (0.015 – 0.272) | **0.029** |

**Table 7. Cox regression model for 5-year patient survival**

HR = hazard ratios, CI = 95% confidence interval. HC = historic cohort, ESP = European Senior Program, BMI = body mass index, HLA = human leukocyte antigen, PF = primary function

**New:**

Table 7

| **Cox regression model for 5-year patient survival** | | | | |
| --- | --- | --- | --- | --- |
| **Parameters** | **Univariate** | | **Multivariable** | |
|  | **HR**  **(95% CI)** | **p-value** | **HR**  **(95% CI)** | **p-value** |
| **HC vs nESPC** | 1.829  (0.734 – 4.557) | 0.195 |  | |
| **Recipient age**  **(years)** | 0.955  (0.870 – 1.049) | 0.337 |  | |
| **Recipient sex**  **(male vs female)** | 2.100  (1.144 – 3.855) | **0.017** | 0.971  (0.634 – 1.489) | 0.422 |
| **Recipient BMI**  **(<25 kg/m^2^)** | 2.225  (0.984 – 5.032) | 0.055 |  | |
| **Time on dialysis**  **(months)** | 1.012  (0.990 – 1.035) | 0.287 |  | |
| **Arterial hypertension recipient** | 0.617  (0.289 – 1.319) | 0.213 |  | |
| **Diabetes recipient** | 2.671  (1.311 – 5.442) | **0.007** | 1.819  (1.132 – 2.921) | 0.090 |
| **Hyperlipidemia recipient** | 0.989  (0.476 – 2.503) | 0.976 |  | |
| **Arteriosclerosis recipient** | 3.152  (1.620 – 6.132) | **0.001** | 1.379  (0.899 – 2.114) | 0.958 |
| **Cold ischemia time**  **(hours)** | 0.911  (0.840 – 0.988) | **0.025** | 0.954  (0.910 – 1.000) | 0.272 |
| **Sum of HLA mismatches** | 0.830  (0.636 – 1.085) | 0.173 |  | |
| **Operation time**  **(min)** | 1.000  (1.000 – 1.000) | **0.010** | 1.000  (1.000 – 1.000) | 0.268 |
| **Warm ischemia time**  **(min)** | 0.987  (0.961 – 1.013) | 0.310 |  | |
| **Intraoperative complications**  **(yes vs no)** | 2.212  (0.823 – 5.945) | 0.116 |  | |
| **Postoperative complications**  **(yes vs no)** | 1.134  (0.652 – 1.972) | 0.656 |  | |
| **Length of hospital stay**  **(days)** | 1.005  (0.986 – 1.025) | 0.606 |  | |
|  |  |  |  | |
| **Donor age**  **(years)** | 0.976  (0.920 – 1.036) | 0.432 |  | |
| **Donor gender**  **(male vs female)** | 1.113  (0.547 – 2.266) | 0.767 |  | |
| **Donor BMI**  **(kg/m^2^)** | 0.988  (0.912 – 1.069) | 0.761 |  | |
| **Donor creatinine pre-procurement (mg/dl)** | 1.118  (0.533 – 2.345) | 0.769 |  | |
| **Arteriell Hypertension Donor** | 1.310  (0.689 – 2.489) | 0.410 |  | |
| **Diabetes Donor** | 1.404  (0.635 – 3.106) | 0.402 |  | |
| **Hyperlipidemia Donor** | 2.157  (0.795 – 5.851) | 0.131 |  | |
| **Arteriosclerosis Donor** | 0.793  (0.405 – 1.553) | 0.499 |  | |
| **Length of Donor on Intensive Care Unit (d)** | 1.083  (1.017 – 1.153) | **0.013** | 1.053  (1.000 – 1.109) | 0.351 |
|  |  |  |  | |
| **PF**  **(yes vs no)** | 0.272  (0.065 – 1.143) | 0.076 |  | |
| **Biopsy-proven rejection**  **(yes vs no)** | 0.707  (0.419 – 1.193) | 0.194 |  | |
| **Intraoperative complications**  **(yes vs no)** | 0.572  (0.297 – 1.102) | 0.095 |  | |
| **Postoperative surgical complications (yes vs no)** | 0.954  (0.634 – 1.436) | 0.822 |  | |
| **Non-surgical complications**  **(yes vs no)** | 0.510  (0.338 – 0.769) | **<0.001** | 0.198  (0.015 – 0.272) | **0.029** |

**Table 7. Cox regression model for 5-year patient survival**

HR = hazard ratios, CI = 95% confidence interval. HC = historic cohort, ESP = European Senior Program, BMI = body mass index, HLA = human leukocyte antigen, PF = primary function
